# Octanoic acid a major component of widely consumed medium-chain triglyceride ketogenic diet is detrimental to bone

**DOI:** 10.1038/s41598-021-86468-9

**Published:** 2021-03-26

**Authors:** Shreshta Jain, Reena Rai, Divya Singh, Divya Vohora

**Affiliations:** 1 Department of Pharmacology, School of Pharmaceutical Education and Research, Jamia Hamdard, New Delhi 110062 India; 2 Endocrinology Division, Central Drug Research Institute (CDRI), Lucknow, India

**Keywords:** Medical research, Rheumatology

## Abstract

Octanoic acid is a medium-chained saturated fatty acid found abundantly in the ketogenic dietary supplements containing medium chained triglycerides (MCT) along with decanoic acid. The MCT ketogenic diet is commonly consumed for weight loss but has also showcased neuroprotective potential against neurodegenerative disorders. However, recent clinical findings have reported a critical disadvantage with the long-term consumption of ketogenic diet i.e. bone loss. The following study was employed to investigate whether the two major components of MCT diet also possess bone loss potential as observed with classical ketogenic diet. Swiss albino mice aged between 10 and 12 weeks, were divided into 3 treatment groups that were administered with oral suspensions of octanoic acid, decanoic acid and a combination of both for 4 weeks. Bone specific markers, microarchitectural parameters, using micro computed tomography, and biomechanical strength were analyzed. Remarkably deleterious alterations in the trabecular bone microarchitecture, and on bone markers were observed in the octanoic acid treated groups. Our results suggest significant negative effects on bone health by octanoic acid. These findings require further investigation and validation in order to provide significant clinically relevant data to possibly modify dietary composition of the MCT ketogenic diet.

## Introduction

Ketogenic diet consists of high fat, low carbohydrates with a moderate protein intake. It is responsible for the production of ketone bodies as an alternate source of energy in humans, a state termed as ketosis. The diet has been modified over the years, with modified Atkin’s diet and the incorporation of medium-chained triglycerides (MCT). The MCTs can be derived from natural sources such as coconut oil and palm tree oil and are composed of octanoic acid (Caprylic acid: 65–75%), decanoic acid (Capric acid: 25–35%), hexanoic acid (Caproic acid: 1–2%) and dodecanoic acid (Lauric acid: 1–2%)^1^. Commercially available over-the-counter MCT supplements are being manufactured by nutritional companies such as Simyl, Luxura Sciences, Kayos Naturals, Healthvit, Nutiva, Grow Fit, etc., in the form of oils and/or powders. The composition of these marketed products includes 60–70% of octanoic acid and 30–40% of decanoic acid, whereby 15–20 g of MCT is recommended as amount of daily intake, as per the product labels. Ketogenic diet is clinically approved for the treatment of intractable epilepsy in children and in adults^2,3^. MCT supplemented type of ketogenic diet (MCT diet) has also been widely used as a treatment against neurodegenerative disorders with promising results in controlling seizure activity in children with refractory epilepsy^4,5^. Clinical evidences has shown remarkable seizure reduction in not only children but also in adults^4–9^ such as the case report of a 43-year old male patient with drug-resistant partial epilepsy after administering MCT oil supplements^7^. MCT supplemented ketogenic diet has also expressed neuroprotective effects by improving the Alzheimer’s disease assessment score in patients during a ketogenic diet retention and feasibility trial conducted by Matthew K. Taylor and his co-workers^10^. MCT diet has also been researched in other metabolic disorders such as cancer and diabetes, on the basis of their various mechanisms of action^11^. Commonly, the short term use of the low caloric MCT diet is associated with weight loss, fat loss and changes in whole body composition including fat mass and adipose tissue distribution referred from clinical and meta-analysis studies performed^12,13^.

The mechanism of prevention of neurodegeneration by MCT is associated with the production of ketone bodies such as β-hydroxybutyrate (BHB)^11^. Among MCT, decanoic acid has exhibited anti-convulsant potential via selective inhibition of glutamatergic AMPA receptors^14^. Anti-inflammatory action of decanoic acid explored on comparison with lauric acid resulted in inhibition of nuclear factor kappa-B (NF-kB) signaling^15^. Decanoic acid also acts an agonist for peroxisome proliferator-activated receptors-PPARγ^16^. The cause of octanoic acid exhibiting anti-convulsant action remains unknown.

Ketogenic diet, although deemed efficacious, has been associated with low bone mineral density according to numerous clinical findings^17,18^. Decanoic acid and octanoic acid, major components of MCT diet, are classified under saturated fatty acids; in vitro analysis of predominantly used saturated fatty acids in everyday diet such as palmitic acid, stearic acid and lauric acid, suggested osteoclastogenesis and development of osteoarthritis^19^. However, pre-clinical investigation of decanoic acid on osteoclasts suggested inhibition of lipopolysaccharide (LPS) and receptor activator of nuclear factor kappa-Β ligand (RANKL)-induced osteoclastogenesis^20,21^. According to these controversial outcomes, there lies a need to investigate the effects of MCT diet on bone health. To the best of our knowledge, no study has evaluated the role of individual components of MCT ketogenic diet to ascertain effects on bone after in vivo administration. Further, no in vitro or in vivo studies have been performed with octanoic acid. The present work investigated the effects of administration of MCT dietary components namely decanoic acid and octanoic acid alone and in combination on the bone micro-architectural parameters, biomechanical strength and turnover markers in Swiss albino mice.

## Materials and methods

### Animals

Male Swiss albino mice, aged between 10 and 12 weeks, were acquired from the on-campus Central animal house facility, Jamia Hamdard, New Delhi after the ethical approval by the Institutional Animal Ethics Committee of Jamia Hamdard (Protocol no.-1513; Year-2018). The average weights of animals prior to the initiation of treatment are mentioned in Table 1. The mice were housed in groups of 6 per cage, with the standard chow diet and water ad libitum, and were allowed to acclimatize for 7 days in laboratory. All the animals were exposed to the same level of stress regardless of their treatment groups. The temperature and humidity were maintained at 25 ± 2 °C and 55–60% respectively along with 12 h of light–dark cycle. The guidelines by the Committee for the Purpose of Control and Supervision of Experiments on Animals (CPCSEA), India were followed. All the information reported in the study is in accordance with the ARRIVE guidelines.

**Table 1:** Mean body weights (g) ± SD of Swiss Albino mice as per the treatment groups at the start of experimental study.

	Treatment groups	Mean body weight ± SD (g)
I	Control	40.88 ± 1.869
II	DA	43.90 ± 3.578
III	OA	42.58 ± 3.475
IV	OA + DA	43.88 ± 4.006

### Experimental design

Swiss albino mice were divided, as per simple randomization, into the following groups each containing 6 animals: Group I (Control): 0.5% Aqueous solution of methyl cellulose (p.o.); Group II (DA): Decanoic acid 1100 mg/kg (p.o.); Group III (OA): Octanoic acid 1700 mg/kg (p.o.); Group IV (OA + DA): Decanoic acid 1100 mg/kg (p.o.) + Octanoic acid 1700 mg/kg (p.o.). The animals were given oral suspensions of octanoic acid and decanoic acid in 0.5% methyl cellulose aqueous solution for a period of 30 days, using oral gavage. The doses were derived from their human equivalent dose (HED) counterparts using allometric scaling calculation based on the body surface area by either dividing or multiplying the human dose (mg/kg) by a correction factor, known as the K_m_ ratio^22^. The sample size was calculated using G*Power software (version 3.1.9.4 for Windows 10) on the basis of previous available data from the investigation of the effects of ketogenic diet on bone turnover markers-ALP and TRAP along with the micro-computed tomography of the distal femur bone in mice^23^ at the power pf 0.97.

### Urine analysis for ketone body

The ketone bodies level was measured, using ketone reagent strips (Keto-Diastix, Bayers) for urinalysis, after completing the 4 weeks duration of oral administration of decanoic acid and octanoic acid.

### Micro-architectural analysis and Bone mineral density analysis using micro-CT

At the end of 4 weeks, the mice were euthanized via CO_2_ inhalation followed by cervical dislocation and the right femur and tibia bones of each animal were excised out and cleaned off of any muscle or tissue. For micro-computed tomography, the bones were scanned using Sky Scan 1076 CT scanner (SkyScan, Aartselaar, Belgium) with pixel size of 9 μm^3^, 0.5 mm filter and the X-rays source set at 50 kV and 200 mA. The total projections captured were 236 at an angular arrange of 360°. The reconstruction in the form of three-dimensional images was performed by SkyScan Nrecon Software. The distal femur and proximal tibia bones for image reconstruction was selected on the basis of extreme bone micro-architecture degradation. Ellipsoid contours were drawn using CT Analyzer (CTAn, SkyScan) in the selected trabecular region of bone. For the analysis of trabecular region, the region of interest (ROI) was drawn at a total of 100 slices in the secondary spongiosa region located at 1.5 mm from the distal border of growth plate for femoral bones and proximally for tibia bones, eliminating all primary spongiosa and cortical bone. To analyze cortical bone, 350 serial images were rejected from the growth plate to remove any trabecular region and the following 100 consecutive images were chosen to perform quantification using CTAN software. The threshold of 50–55% was selected by positioning the threshold lines to be clear of noise fluctuation near the central region (vertically) of the display. The micro-architectural parameters documented were: trabecular bone volume (BV/TV, %), trabecular thickness (Tb.Th, mm), trabecular separation (Tb.Sp, mm), trabecular number (Tb.N, 1/mm), and trabecular pattern factor (Tb.Pf, 1/mm), mean total cross-sectional bone area (B.Ar), mean total cross-sectional tissue area (T.Ar), and cross-sectional thickness (Cs.Th).

The bone mineral density (BMD, g/cm^2^) was also determined from which bone mineral content (BMC) was calculated using the formula BMC = BMD X bone volume (BV)^24^. The analysis was performed and reported according to the guidelines provided by the American Society for Bone and Mineral Research^25^.

### Mechanical strength test

Bone mechanical strength assay was performed by the 3-point bending strength of femur mid-diaphysis using Bone Strength Tester Model TK-252C (Muromachi Kikai Co. Ltd., Tokyo, Japan). The distance between the supports was fixed constant at 1 cm with a displacement rate of 0.03 mm/s. The load–displacement curves generated were used to calculate the ultimate load (N), stiffness (N/mm), and energy to fracture (N-mm).

### Bone ALP and TRAP levels

The femoral bones were later crushed and then homogenized with 10 volumes of phosphate buffer saline (pH-7.4). The homogenates were centrifuged at 2000–3000 rpm for 20 min. The aliquots of the bone extracts were used for biochemical investigations. Bone formation marker, alkaline phosphatase^25^ was estimated using commercially purchased diagnostic kit from Arkray, India, and bone resorption marker tartrate‑resistant acid phosphatase (TRAP5b) following the procedure provided by Tenniswood and co-workers^26^.

### Statistical analysis

The data was analyzed using one-way ANOVA followed by Tukey Kramer multiple comparison tests. Data was represented as mean ± standard deviation. All statistical tests were performed using the Prism software package (version 8, GraphPad, San Diego, CA). *p* < 0.05 was considered to be significant.

## Ethical approval

We confirm that we have read the Journal’s position on issues involved in ethical publication and affirm that this report is consistent with those guidelines.

## Results

### Urinary ketone body level on oral administration of octanoic acid and decanoic acid

**Figure 1:**
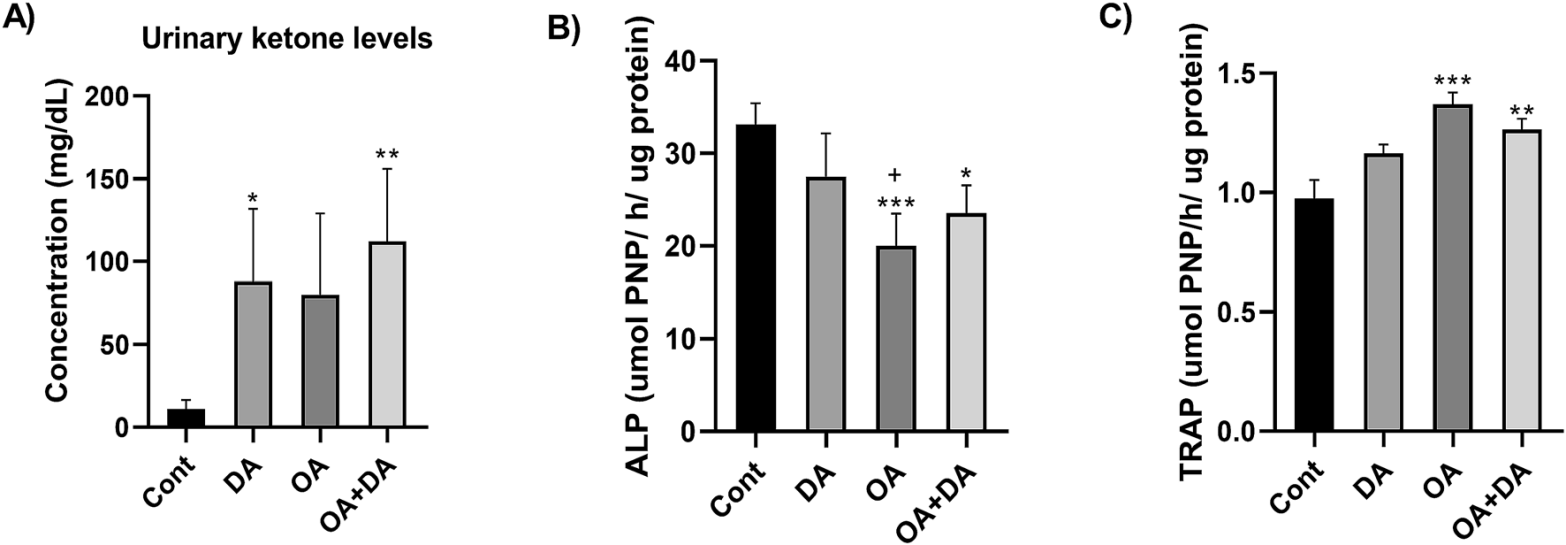
The figure shows the effects of decanoic acid, octanoic acid and their combination on the (**A**) ketone bodies detected on urine analysis, and biochemical estimation of (**B**) bone specific alkaline phosphatase (ALP) levels and (**C**) bone specific tartarate-resistant acid phosphatase (TRAP) levels in femur bones of mice. Values are represented as mean ± SD and analyzed by one-way ANOVA followed by Tukey Kramer multiple comparison test. The significance is ascertained as ***p < 0.001; **p < 0.01; *p < 0.05 versus Cont; +*p* < 0.05 versus DA. Cont-Control; DA—Decanoic acid; OA—Octanoic acid.

On comparing with the control group, that were only fed with standard chow diet, an increase in the ketone bodies concentration is depicted in Fig. 1A in MCT treated groups, determined via urinalysis. Oral administration of decanoic acid displayed a significant rise (*p* < 0.05 versus control) in the ketone body levels at the end of 4 weeks of intervention. A higher increase was observed when both decanoic acid and octanoic acid were administered in combination (*p* < 0.01 versus control). The mean body weights at the end of 4 weeks are given in Table 2.

**Table 2:** Mean body weights (g) ± SD of Swiss Albino mice as per the treatment groups at the end of 4 weeks of experimental study.

	Treatment groups	Mean body weight ± SD (g)
I	Control	39.5 ± 1.75
II	DA	37.4 ± 1.71
III	OA	32.0 ± 3.85
IV	OA + DA	34.3 ± 4.29

### ALP and TRAP levels in bone specific biochemical assays

The results are shown in Fig. 1B,C. Alkaline phosphatase is widely considered as the bone formation marker and it was observed to be significantly reduced upon oral administration of octanoic acid (*p* < 0.001 versus control). Furthermore, there was also a significant difference in the levels of ALP, between the octanoic acid treated group and the groups treated with decanoic acid (*p* < 0.05 vs. DA). Concordantly, octanoic acid was also responsible in significantly elevating the levels of TRAP (*p* < 0.001 versus control). On the contrary, decanoic acid exhibited no significant effect on either of the markers.

### Bone mineral density and Micro-architectural analysis of trabecular region of femur and tibia bones^*^

**Figure 2:**
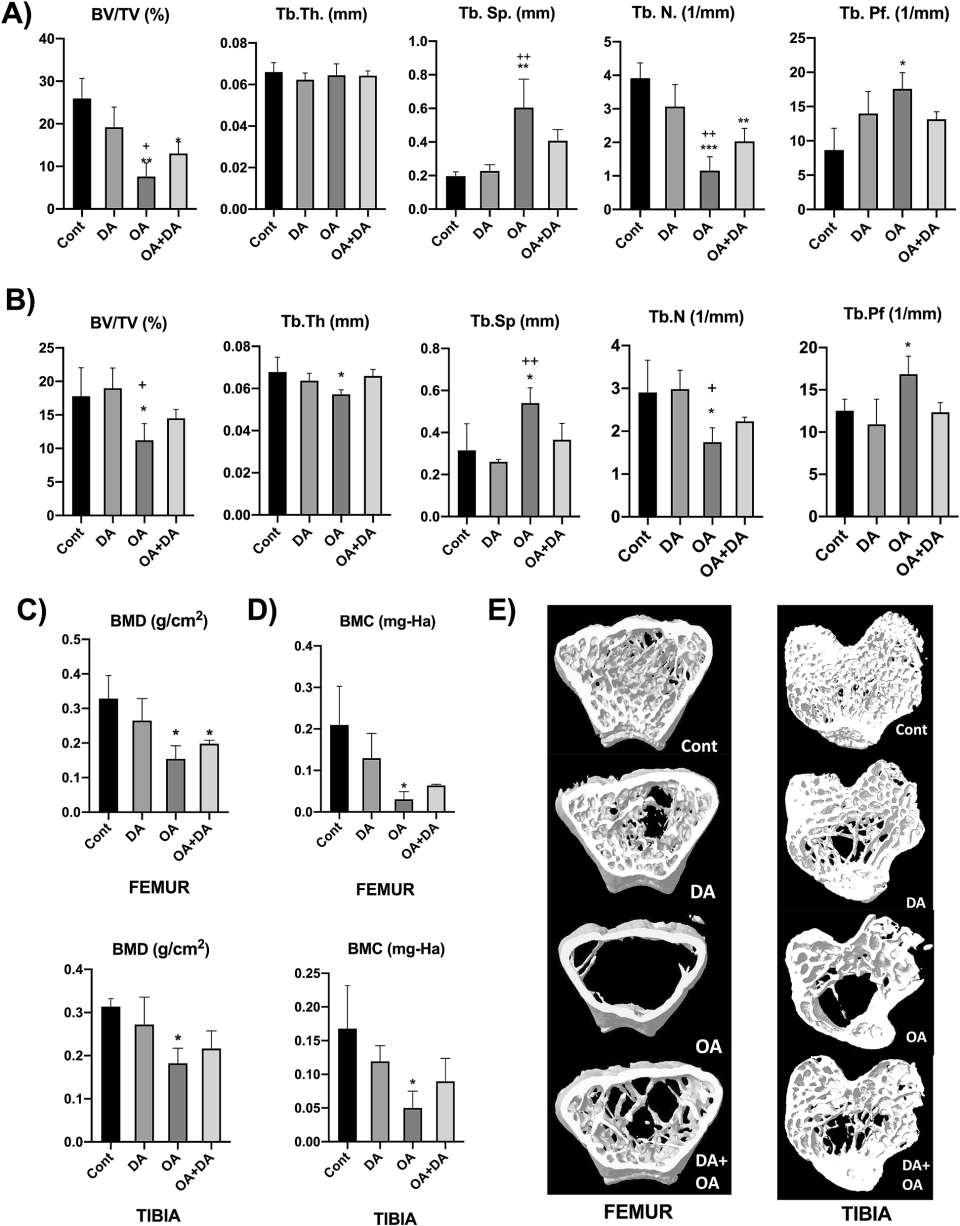
Effect of decanoic acid, octanoic acid and their combination on microcomputed tomography analysis including trabecular bone volume (BV/TV, %), trabecular thickness (Tb.Th, mm), trabecular separation (Tb.Sp, mm), trabecular number (Tb.N, 1/mm), and trabecular pattern factor (Tb.Pf, 1/mm) in both (**A**) femur bone and (**B**) tibia bone, and on (**C**) bone mineral density (BMD, g/cm^2^) along with (**D**) bone mineral content (BMC, mg-HA) in femur and tibia bones. (**E**) The figure shows three-dimensional reconstructed images of the trabecular region of femur and tibia bones. Values are represented as mean ± SD and analyzed by one-way ANOVA followed by Tuckey Kramer’s multiple comparison test. The significance is ascertained as ***p < 0.001; **p < 0.01; *p < 0.05 versus Cont; ++*p* < 0.001; +*p* < 0.05 versus DA. Cont-Control; DA- Decanoic acid; OA- Octanoic acid.

^*^The results obtained from the micro-architectural analysis of the cortical bone for parameters including mean total cross-sectional bone area (B.Ar), mean total cross-sectional tissue area (T.Ar), and cross-sectional thickness (Cs.Th), were non-significant and hence, has not been shown in this study and are provided as Supplementary materials.

The findings are presented in Fig. 2. Both the trabecular bone mineral density (BMD) and the bone mineral content (BMC) were observed to be significantly lower only in groups treated with octanoic acid in both femur and tibia bones (*p* < 0.05 versus control) (Fig. 2C,D). The trabecular bone volume and the trabecular number were also observed to follow the similar significant reduction in both femur and tibia bones as compared to control and decanoic acid. Conversely, the trabecular separation and pattern factor were markedly enhanced in the groups treated with octanoic acid when compared to control. Also, there was an increase in the trabecular separation with octanoic acid in comparison to decanoic acid. Thus, while the analysis of groups treated with decanoic acid presented non-significant results; however, octanoic acid treated groups when compared with decanoic acid treated groups, resulted in significant differences in the microarchitectural parameters of both femoral and tibial bones (*p* < 0.05, *p* < 0.01 vs. DA). Interestingly, the combined treatment group exhibited no remarkable effects. No alteration was observed in the trabecular thickness of femur bones (Fig. 2A), however a significant reduction was observed in tibia bones (Fig. 2B). The alterations in the trabecular microarchitecture by octanoic acid are remarkably visible in the reconstructed 3-D images of the femur as well as in tibia bones as depicted in Fig. 2E.

### Bone biomechanical strength

The mechanical strength test of the cortical region of the femur bones resulted with no significant outcomes, hence has not been showcased here, but can be viewed under Supplementary Materials.

## Discussion

The consumption of MCT has been globally accepted as a dietary treatment against not only refractory epilepsy in children as well as adults but also against a number of other neurodegenerative disorders and metabolic disorders^11^ in addition to the over the counter use to reduce body weight^13^. Here, we report for the first time that octanoic acid, one of the major components of MCT ketogenic diet, is detrimental to bone and requires caution in patients susceptible to bone depletion. Given the fact that both epilepsy and antiepileptic drug therapy have been linked to deteriorating effects on bone^27^ and that the ketogenic MCT diet is widely consumed, the findings of our study may have significant translational relevance for persons with epilepsy and population in general.

We observed that octanoic acid treatment for 30 days reduced the levels of alkaline phosphatase (ALP), a marker for bone formation and increased the levels of tartrate resistant acid phosphatase (TRAP), a bone resorption marker in bones. These findings were further substantiated by micro-architectural analysis of trabecular part of femoral and tibial bones where octanoic acid treated mice elicited remarkable alterations showing maximum deleterious effects in three-dimensional reconstructed images along with the evident reduction in both, bone mineral density and bone mineral content, confirming the adverse effects on bone due to octanoic acid treatment. Therefore, the results obtained in our study provide firm evidence that octanoic acid exhibit detrimental effects on bone mineralization.

On the contrary, decanoic acid illustrated no remarkable effects. The results suggested that the involvement of decanoic acid in degrading bone strength is minimal and could be explained by the results obtained from previous in vitro investigations implemented by Park et al. and Kim et al^20,21^ where decanoic acid exhibited inhibition against lipopolysaccharide (LPS)-induced osteoclastogenesis and receptor activator of nuclear factor κB (RANKL)-induced differentiation in mature osteoclasts, respectively. However, no such in vitro data is available for octanoic acid. These findings can also be substantiated with evidence pertaining to greater oxidation of octanoic acid than decanoic acid leading to the accumulation of the latter in the body^28^, thereby, suggesting the impact of increased production of oxidative metabolites of octanoic acid on bone resorption.

The ketosis can be induced either naturally by reduction in carbohydrate intake or caloric intake or could be independent of diet simply by administration of medium chained triglycerides. In our study too, the urinary ketone levels were raised only in groups that were administered with medium chained fatty acid- decanoic acid and octanoic acid, for inducing ketosis. While the exact mechanisms behind the bone resorption potential shown by octanoic acid ingestion remains unknown, the role of ketone bodies appears unlikely from our results. Though it has been postulated that the ketone bodies are regulated in osteoclast cells in obesity model induced by high fat diet consumption^29^, there are investigations that suggest a positive role of the ketone body, beta-hydroxybutyrate (BHB) on the proliferation of osteoblasts as well as bone growth promoting effects in ovariectomized rats^30^. In addition, anti-osteoporotic actions of BHB has been reported in mice with degraded bone density due to stimulated microgravity^31^. These contradictory outcomes resulted in the probability of an alternate mechanism other than the production of ketone bodies, for the negative impact on bone health caused by octanoic acid requiring further investigations. Another point that needs to be reaffirmed is that all animals were fed normal chow diet concurrently with the fatty acid treatment that contains about 50% of carbohydrate that can possibly counteract some ketosis causing bone depletion. Moreover, the effects on urinary ketone body was lowest for octanoic acid treated group. Therefore, our findings suggest that the bone depletion depicted by octanoic acid in our study was not due to ketosis.

Our study has its limitations. The information gathered from this study remains partial as only the trabecular region of the bones provided significant data; in order to confirm the bone resorptive potential of octanoic acid, the evaluation of cortical regions of bones is also required. In our study, the analysis of cortical bones boomed no significant result and hence, were inconsequential. However, we believe this was due to the short duration of treatment with MCT components and propose further investigation with long term administration of both decanoic acid and octanoic acid to evaluate the effects on cortical bone as well. Furthermore, the impact of physical activity or exercise on bone health while consuming MCT ketogenic diet was not partaken into the objectives of this study. This factor has become crucial after recent findings that suggest short term intake of low carbohydrate ketogenic diet has resulted in impairment of bone health in athletic volunteers^32^. The outcomes of our study regardless of its limitations, implies the need of clinical investigations to be employed in order to validate the detrimental effects of ketogenic diet including MCT diet.

To conclude, we report a significant negative effect on bone by octanoic acid, one of the major components of MCT ketogenic diet. We propose an urgent need to investigate the individual components of the diet for potential adverse effects and to modify dietary MCT ketogenic diet composition accordingly. This is important in view of medium chain triglycerides being endorsed globally as a dietary treatment against neurodegenerative disorders such as refractory epilepsy and Alzheimer’s disease, both of which have been associated with bone deficits.

## Supplementary Information

Supplementary Information

## Data Availability

The datasets generated during and/or analyzed during the current study are available from the first and corresponding author on reasonable request.
