# Supplementary material for: Octanoic acid a major component of widely consumed medium-chain triglyceride ketogenic diet is detrimental to bone
Source: Sci Rep. 2021 Mar 26;11:7003. doi: 10.1038/s41598-021-86468-9 (PMC7997977; doi:10.1038/s41598-021-86468-9)
Supplement: Supplementary file 1 — Supplementary Information [file 41598_2021_86468_MOESM1_ESM.docx]

**Octanoic acid, one of the major components of widely consumed medium-chain triglyceride ketogenic diet, is detrimental to bone**

Shreshta Jain^1^, Reena Rai^2^, Divya Singh^2^, Divya Vohora^1*^

^1^Department of Pharmacology, School of Pharmaceutical education and research, Jamia Hamdard, New Delhi, India

^2^ Endocrinology Division, Central Drug Research Institute (CDRI), Lucknow, India

**SUPPLEMENTARY MATERIALS**

**RESULTS:**

***Micro-architectural analysis of cortical region of femur and tibia bones-***

The results obtained from the micro-architectural analysis of the cortical bone were insignificant.

Table 1-

Effect of decanoic acid, octanoic acid and their combination on cortical region of FEMUR bones:

| **Groups** | **Drug treatment** | **Cortical bone area (Ct.Ar, mm^2^)** | **Mean total cross-sectional tissue area (Tt.Ar, mm^2^)** | **Cortical thickness (mm)** |
| --- | --- | --- | --- | --- |
| I | Control | 0.780 ± 0.0748 | 0.784 ± 0.0774 | 0.165 ± 0.0039 |
| II | DA | 0.915 ± 0.1142 | 0.919 ± 0.1150 | 0.189 ± 0.0170 |
| III | OA | 0.791 ± 0.1074 | 0.792 ± 0.1080 | 0.171 ± 0.0188 |
| IV | DA + OA | 0.854 ± 0.8544 | 0.856 ± 0.0601 | 0.174 ± 0.0093 |

The table shows data of cortical bone parameters- Cortical bone area (Ct.Ar, mm^2^); Mean total cross-sectional tissue area inside the periosteal envelope (Tt.Ar, mm^2^); and Cortical thickness (mm). Values are represented as Mean ± SD and analyzed by one-way ANOVA followed by Tukey Kramer multiple comparison test.

DA- Decanoic acid, OA- Octanoic acid

Table 2-

Effect of decanoic acid, octanoic acid and their combination on cortical region of TIBIA bones:

| **Groups** | **Drug treatment** | **Cortical bone area (Ct.Ar, mm^2^)** | **Mean total cross-sectional tissue area (Tt.Ar, mm^2^)** | **Cortical thickness (mm)** |
| --- | --- | --- | --- | --- |
| I | Control | 0.726 ± 0.0664 | 0.742 ± 0.0664 | 0.148 ± 0.0058 |
| II | DA | 0.857 ± 0.213 | 0.870 ± 0.218 | 0.160 ± 0.0206 |
| III | OA | 0.685 ± 0.0805 | 0.698 ± 0.0767 | 0.134 ± 0.0109 |
| IV | DA+ OA | 0.848 ± 0.0456 | 0.864 ± 0.0406 | 0.156 ± 0.0211 |

The table shows data of cortical bone parameters- Cortical bone area (Ct.Ar, mm^2^); Mean total cross-sectional tissue area inside the periosteal envelope (Tt.Ar, mm^2^); and Cortical thickness (mm). Values are represented as Mean ± SD and analyzed by one-way ANOVA followed by Tukey Kramer multiple comparison test.

DA- Decanoic acid, OA- Octanoic acid

***Mechanical Strength Test-***

The mechanical strength test of the cortical region of the femur bones resulted with no significant outcomes.

Table 3-

Effect of decanoic acid, octanoic acid and their combination on femoral bone strength:

| **Groups** | **Drug treatment** | **Ultimate load (N)** | **Energy to failure (mJ)** | **Stiffness (N/mm)** |
| --- | --- | --- | --- | --- |
| I | Control | 31.83 ± 2.269 | 246.0 ± 2.588 | 0.30 ± 0.141 |
| II | DA | 32.55 ± 3.291 | 245.8 ± 1.679 | 0.20 ± 0.129 |
| III | OA | 27.78 ± 1.987 | 244.5 ± 2.798 | 0.14 ± 0.057 |
| IV | DA+ OA | 30.25 ± 1.457 | 244.3 ± 3.511 | 0.18 ± 0.081 |

The figure shows data of femoral bone strength parameters- ultimate load (N), energy to failure (mJ) and stiffness (N/mm) Values are represented as Mean ± SD and analyzed by one-way ANOVA followed by Tukey Kramer multiple comparison test.

DA- Decanoic acid, OA- Octanoic acid
